# A Two-Gene Signature for Tuberculosis Diagnosis in Persons With Advanced HIV

**DOI:** 10.3389/fimmu.2021.631165

**Published:** 2021-02-22

**Authors:** Vandana Kulkarni, Artur T. L. Queiroz, Shashi Sangle, Anju Kagal, Sonali Salvi, Amita Gupta, Jerrold Ellner, Dileep Kadam, Valeria C. Rolla, Bruno B. Andrade, Padmini Salgame, Vidya Mave

**Affiliations:** ^1^Byramjee-Jeejeebhoy Government Medical College-Johns Hopkins University Clinical Research Site (BJGMC-JHU CRS), Pune, India; ^2^Instituto Gonçalo Moniz, Fundação Oswaldo Cruz, Salvador, Brazil; ^3^Multinational Organization Network Sponsoring Translational and Epidemiological Research (MONSTER) Initiative, Salvador, Brazil; ^4^Johns Hopkins University School of Medicine, Baltimore, MD, United States; ^5^Rutgers- New Jersey Medical School, Center for Emerging Pathogens, Newark, NJ, United States; ^6^Instituto Nacional de Infectologia Evandro Chagas, Fundação Oswaldo Cruz, Rio de Janeiro, Brazil

**Keywords:** HIV, tuberculosis, transcriptomics, diagnosis, gene signature

## Abstract

**Background:** Transcriptomic signatures for tuberculosis (TB) have been proposed and represent a promising diagnostic tool. Data remain limited in persons with advanced HIV.

**Methods:** We enrolled 30 patients with advanced HIV (CD4 <100 cells/mm^3^) in India; 16 with active TB and 14 without. Whole-blood RNA sequencing was performed; these data were merged with a publicly available dataset from Uganda (*n* = 33; 18 with TB and 15 without). Transcriptomic profiling and machine learning algorithms identified an optimal gene signature for TB classification. Receiver operating characteristic analysis was used to assess performance.

**Results:** Among 565 differentially expressed genes identified for TB, 40 were shared across India and Uganda cohorts. Common upregulated pathways reflect Toll-like receptor cascades and neutrophil degranulation. The machine-learning decision-tree algorithm selected gene expression values from *RAB20* and *INSL3* as most informative for TB classification. The signature accurately classified TB in discovery cohorts (India AUC 0.95 and Uganda AUC 1.0; *p* < 0.001); accuracy was fair in external validation cohorts.

**Conclusions:** Expression values of *RAB20* and *INSL3* genes in peripheral blood compose a biosignature that accurately classified TB status among patients with advanced HIV in two geographically distinct cohorts. The functional analysis suggests pathways previously reported in TB pathogenesis.

## Introduction

Tremendous advances in tuberculosis diagnosis have been made based on nucleic acid amplification of bacteria in the sputum, such as Xpert MTB/RIF sputum smear and culture, which provides results in 2 h (1–5). However, sputum-based diagnostics remain problematic in the context of HIV infection. Sputum smear is often negative for TB bacilli, and the sensitivity of Xpert

MTB/RIF is only 67% (6–8). Persons living with advanced HIV (CD4 < 100 cells/mm^3^) are at particularly high risk for TB and are likely to have smear-negative pulmonary or extrapulmonary TB, underscoring the need for non-sputum-based TB diagnostics to support TB control efforts (9–15).

Blood-based transcriptomic signatures, including several parsimonious gene signatures, have been proposed to diagnose and differentiate TB from other respiratory diseases (ORD) and are in various stages of validation (14, 16, 17). However, the majority of studies do not include persons living with advanced HIV. A recent case-control study from Uganda found that transcript levels of *FcGR1A* and *BATF2* and plasma protein levels of interferon gamma (IFN-γ) and CXCL10 were individually accurate classifiers of active TB in the context of advanced HIV (18). However, geographic differences may exist and could impact performance when transcriptomic profiles developed in one population are applied to other geographically distinct populations.

To address the potential influence of geography and the reduced number of TB gene expression signatures addressing persons living with HIV (PLWH), we established a discovery cohort comprising the publicly available RNA sequencing (RNA-seq) dataset from the aforementioned Uganda case-control study (*n* = 33) (18) and RNA-seq data from our prospective case-control study in India among persons with advanced HIV with or without active TB (*n* = 30). Using transcriptomic profiling and a machine-learning approach, we aimed to develop and validate a gene signature to fairly classify TB status among persons with advanced HIV from geographically distinct sites.

## Methods

### Discovery Cohorts

#### India Cohort

Between January 2018 and June 2019, we enrolled 30 consecutive adults attending the antiretroviral treatment (ART) clinic at Byramjee Jeejeebhoy Government Medical College (BJGMC) and Sassoon General Hospitals (SGH), which provides HIV care to residents of Pune, India, and the surrounding area. Eligibility criteria were ART-naïve and ART-experienced adults (>18 years) with advanced HIV, defined as CD4 < 100 cells/mm^3^, with or without newly diagnosed active TB. Exclusion criteria were previous history of TB or anti-tuberculosis treatment (ATT) before enrolment. All potential participants underwent TB symptom screen and GeneXpert MTB/RIF, sputum smear and culture. Cases (TB-HIV), defined as any positive microbiologic TB investigations or ATT initiation based on high clinical suspicion (active TB), were enrolled up to *n* = 15; controls (HIV-only), defined as no evidence of active TB, were enrolled up to *n* = 15. Medical, demographic, socio-economic characteristics, and chest radiograph were obtained at enrolment, and blood samples were collected at baseline for HIV quantitative RNA and CD4+ T-cell count. Individual participant consent as well as BJGMC ethics committee and Johns Hopkins University institutional review committee approvals were obtained.

#### Uganda Cohort

A published case-control study conducted among 33 adults with advanced HIV (CD4 count < 100 cells/mm^3^) in Uganda. The study population comprised 18 cases with active TB (TB-HIV; 16 with smear-positive or microbiologically-confirmed TB and 2 undergoing ATT) and 15 controls (HIV-only) with no clinical symptoms of TB. All participants underwent whole-blood RNA sequencing (RNA-seq) and plasma cytokine/chemokine analysis (18).

### Whole Blood Sample Processing and RNA Sequencing

At enrolment, whole blood (5 mL) was collected from all 30 India participants in two PAXgene Blood RNA tubes (Qiagen, catalog #762165) and directly frozen at −80°C. RNA was extracted using the PAXgene Blood RNA kit (Qiagen, catalog #762174) and quantified using Qubit RNA assay HS (Invitrogen, Cat #Q32852). RNA purity was checked using QIAxpert, and RNA integrity was assessed on TapeStation using RNA HS ScreenTapes (Agilent, Cat #5067-5579). NEB Ultra II Directional RNA-Seq Library Prep kit protocol was used to prepare libraries for total RNA sequencing. Prepared libraries were quantified using Qubit High Sensitivity Assay (Invitrogen, Cat #Q32852), pooled and diluted to final optimal loading concentration before cluster amplification on Illumina flow cell. Once the cluster generation was completed, the cluster flow cell was loaded on Illumina HiSeqX instrument to generate 150bp paired-end reads.

### Gene Expression Analysis

Raw RNA-seq data from the India cohort were retrieved from Illumina HiSeqX in fastq formatted files and processed using the protocol for paired-end reads in the quality check and mapping step; raw RNA-seq data from the Uganda cohort were downloaded from the NCBI SRA database using sra-tools (https://ncbi.github.io/sra-tools/fastq-dump.html) and processed using the single-end protocol in the quality check and mapping step. Low quality bases were removed from all samples, and adapters were trimmed using Trimmomatic V0.32 (19). A total of 5 samples failed in the quality check process from India Cohort and were removed from analysis. A total of 58 samples from both sites were used in downstream analysis. After the quality check, sequences were aligned to the human transcriptome (GRCh38 version 100), comprising mRNA and ncRNA, using Salmon v1.2.0 (20). After the mapping step, the Salmon output was converted to count tables using the tximport R package (21). Count gene expression matrix was examined using the DESeq2 R package (22) to identify differentially expressed genes (DEG) for cases. Changes in gene expression with false discovery rate (FDR)-adjusted *p*-value <0.05 and log_2_fold-change ±1.4 were considered significant. Candidate DEGs were visualized using volcano plots and Venn diagrams using the VennDiagram R package and scanned with the REACTOME pathway database (23) using the compareCluster R package (24). The entire gene expression data set from India cohort is available at the GEO database (Accession number GSE162164, https://www.ncbi.nlm.nih.gov/geo/query/acc.cgi?acc=GSE162164).

### Machine Learning Approach

Following variance-stabilizing transformation and batch effect correction [sva package (25)], gene expression measurements were used to perform a machine learning approach. Using the rpart R package (26), a decision-tree algorithm with leave-one-out cross-validation was applied to identify the minimal variable set (gene set) exhibiting higher classification power to describe cases. The resulting genes were retrieved from each dataset. Sample clustering and classification were assessed using Heatmaps and the Principal component analysis (PCA) plot and applied to the variance-stabilizing transformed gene expression values from each cohort.

### Signature Performance Analysis

We conducted a performance comparison using 36 previously published gene expression signatures for TB diagnosis, progression and treatment provided by the TBSignatureProfiler package (https://github.com/compbiomed/TBSignatureProfiler). In addition, we have included Risk6 signature cohort for comparison (27) (Supplementary Table 1). We applied a general linear model to gene expression values from each signature gene. The outcomes were binarized to measure the sensitivity and specificity of classification, allowing us to measure each group rate and plot area under the curve (AUC) values to identify the best classifier.

### Validation of the Gene Signature

To validate the gene signature, we applied the gene expression model to gene expression data, which was log 2 normalized, from three independent and publicly available patient cohorts (28–30). The first study developed and validated transcriptomic signatures to distinguish TB from latent TB infection (LTBI) using a case-control design among African adults with and without HIV (28); validation was performed by comparing TB-HIV (with and without culture-confirmed TB) vs. HIV-only. The second study identified and validated transcriptomic signatures to distinguish active TB from other respiratory diseases as well as LTBI among large pediatric cohorts from South Africa, Malawi and Kenya (29); the comparison for validation was TB-HIV vs. HIV and other respiratory diseases.

### Statistical Analysis

All analyses were pre-specified. Clinical data were compared among cases and controls using the Mann-Whitney *U* test (continuous variables) or Pearson's chi-square test (categorical variables). Correlations between gene expression and clinical variables were tested using Spearman's rank correlation coefficient. Receiver Operator Characteristics (ROC) were used to assess the accuracy of a gene signature to distinguish between comparison groups specified in the India/Uganda datasets and each validation dataset (*in-silico* validation cohorts). We measured the z-scores with the scales function. Analyses were performed using the base package from R 4.0.2. Differences with *p*-values <0.05 were considered statistically significant.

## Results

### Description of Discovery Cohorts

Cases (*n* = 16) and controls (*n* = 14) from the India cohort (*n* = 30) did not significantly differ among baseline characteristics, including sex (82% male vs. 71% male), median age (45 vs. 41 years), median CD4 count (45 vs. 53 cells/mm^3^) and median HIV viral load (5.50 vs. 4.92 log copies/mL) (Table 1). The Uganda cohort (*n* = 33) was 62% female, median age was 32 years and median CD4 count was 50 cells/mm^3^ with no significant differences between cases (*n* = 18) and controls (*n* = 15) (18).

**Table 1:** Baseline characteristics among cases (TB-HIV) and controls (HIV-only) enrolled in the India cohort (*n* = 30).

**Characteristic**	**HIV-only (*n* = 14)**	**TB-HIV (*n* = 16)**	***p*-value**
**Sex**			
Female, n (%)	4 (29%)	3 (18%)	0.68
Male, n (%)	10 (71%)	13 (82%)	
Median age, y (IQR)	41 (31–52)	45 (38–52)	0.42
**Smoker, n (%)**			
Never	10 (71%)	11 (69%)	>0.95
Former	0	1 (6%)	
Current	4 (29%)	4 (25%)	
Body mass index, kg/m^2^	20.0 (16.8–21.4)	17.6 (16.4–19.9)	0.23
Median HIV viral load, log_10_ copies/mL	4.92 (4.24–5.77)	5.50 (4.97–5.87)	0.32
Median CD4 count, cells/mm^3^ (IQR)	53 (32–75)	48 (31–65)	0.60
Median CD8 count, cells/mm^3^ (IQR)	645 (320–861)	430 (244–589)	0.13
Median CD3 count, cells/mm^3^ (IQR)	739.5 (407–1,043)	491 (290–679.5)	0.15

*BMI, body mass index; HIV, human immunodeficiency virus; TB, tuberculosis*.

### Gene Expression Analysis

A total of 565 DEGs were identified for cases (active TB) among the discovery cohorts. Of these, the majority (488 DEGs) were specific to the Uganda cohort, including 265 upregulated and 223 downregulated genes; 37 were specific to the India cohort, including 32 upregulated and 5 downregulated genes; and 40 were shared by both cohorts (Supplementary Figure 1, Supplementary Table 2). Cluster analysis revealed that DEGs identified at each site were able to distinguish samples from cases and controls, but with some misclassifications (Supplementary Figure 2).

**Figure 1:**
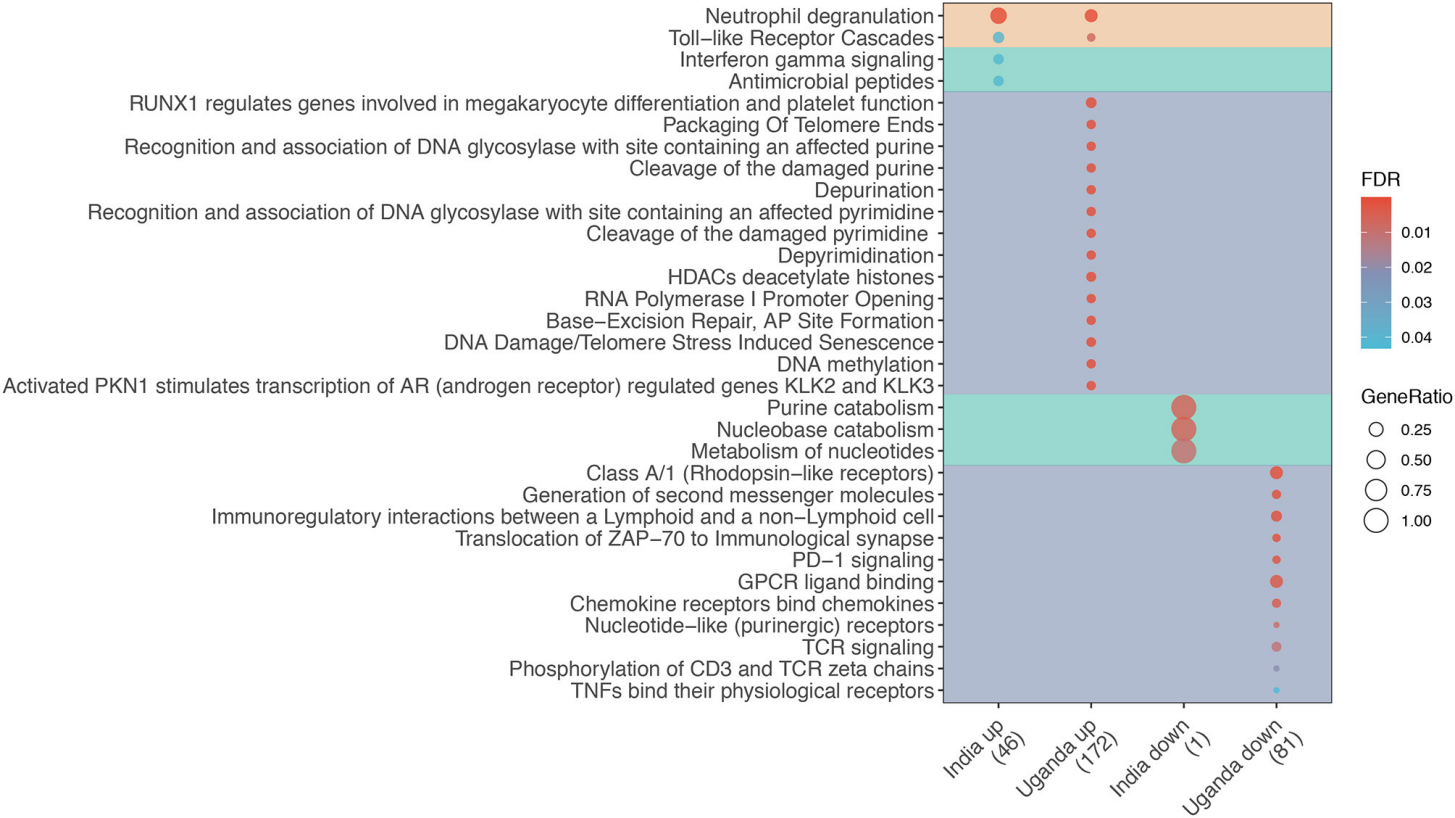
Enrichment analysis of differentially expressed genes (DEG) for TB among adults living with advanced HIV by study site. Dot diameter represents the gene ratio for each pathway, and fill color represents the false discovery rate (FDR)-adjusted *p*-value for the change in gene expression. Shading identifies shared (orange), India-specific (green), and Uganda-specific (purple) pathways.

The majority of shared DEGs were upregulated (38 upregulated vs. 2 downregulated). The enrichment analysis shown in Figure 1 reveals that only two pathways were enriched in both discovery cohorts, namely Toll-like receptor cascades and Neutrophil degranulation. Among Uganda-specific DEGs, upregulated pathways predominantly reflect DNA repair and regulation, and downregulated pathways reflect immune cell response regulation. In contrast, India-specific upregulated pathways reflect IFN-γ signaling and antimicrobial peptide response while downregulated pathways reflect nucleotide metabolism.

### Machine Learning

**Figure 2:**
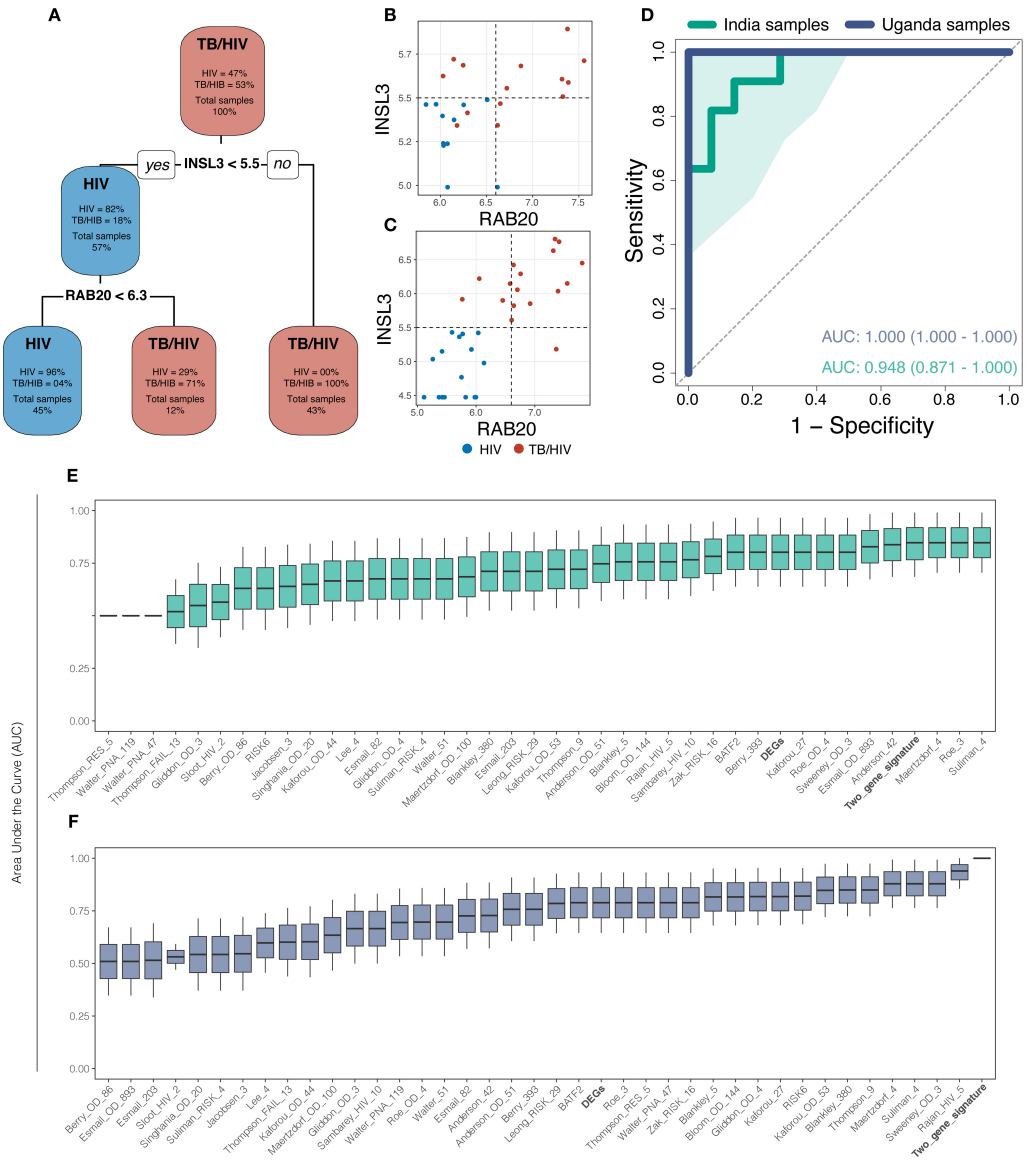
The machine learning approach identified a 2-gene signature (INSL3 and RAB20) that best classified tuberculosis status across study sites. **(A)** The decision-tree algorithm selected INSL3 and RAB20 genes to classify tuberculosis status among the discovery cohorts. **(B,C)** Dot plots show that Decision-tree genes correctly classify TB status for most samples from the India cohort **(B)** and for 100% of samples from the Uganda cohort **(C)**; vertical and horizontal dotted lines represent decision thresholds for RAB20 and INSL3 genes, respectively. **(D)** Receiver operating characteristic (ROC) curve analysis shows strong TB classification performance of Decision-tree genes among samples from India (green line) and Uganda (purple line) with area under the curve (AUC) of 0.948 and 1.00, respectively; shaded area represents standard deviation. Boxplots show the AUC, measured by general linear modeling, for Decision-tree genes (Bold), differentially expressed genes (Bold), and publicly available TB gene expression signatures identifying the Decision-tree genes as the best TB classifier across India **(E)** and Uganda **(F)** cohorts.

Gene expression values from DEGs were used to perform machine learning. The decision tree identified INSL3 and RAB20 (Decision-tree genes) as the optimal gene set to classify tuberculosis status among patients from both sites (Figure 2A). Dot plots show that threshold gene expression values for *INSL3* and *RAB20* fairly classified samples from both study sites, correctly classifying 100% of Uganda samples and returning only 3 classification errors in the India cohort (Figures 2B,C). Receiver operator characteristic (ROC) analysis indicates accurate TB classification among samples from India [AUC 0.95 (0.87–1.00)] and Uganda (AUC 1.00) (Figure 2D). Compared to DEGs and 36 proposed TB gene expression signatures, the Decision-tree genes best classified TB status among samples from both cohorts (Figures 2E,F). Although the Maertzdorf_4, Roe_3 and Suliman_4 signatures and Decision-tree genes performed comparably in the India cohort, the Maertzdorf and Suliman signatures comprises 4 genes and Roe signature comprises 3 genes, and was not as accurate in the Uganda cohort where the Rajan_HIV_5 and Decision-tree signatures performed best. Reviewing potential associations between Decision-tree genes and previously proposed TB signatures revealed that *RAB20* is included in the Bankley_380 (383 genes) and Barry_393 (290 genes) signatures (Supplementary Figure 3A), yet the Decision-tree genes had superior performance in both cohorts.

### Correlation of Clinical Variables With Decision-Tree Gene Expression

**Figure 3:**
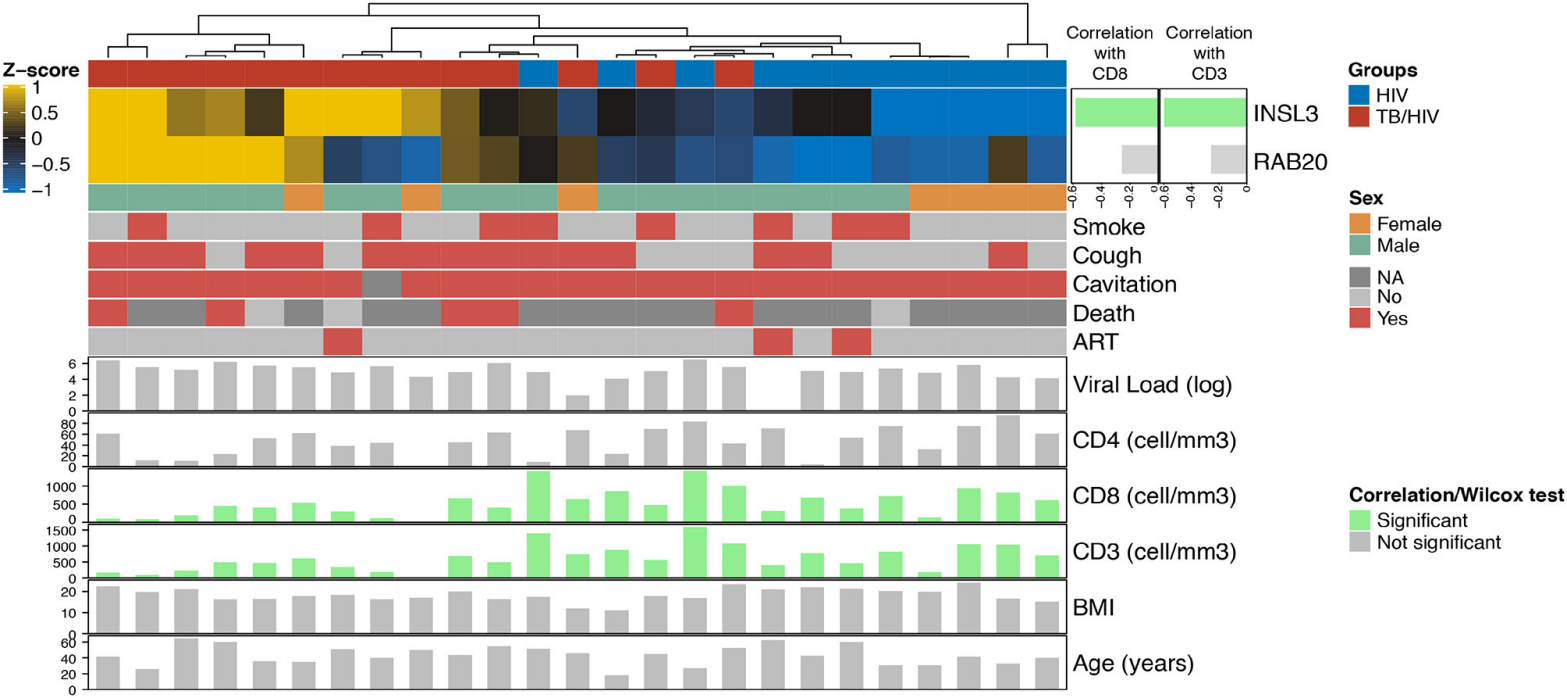
Heatmap showing the relationship between Decision-tree gene expression and clinical characteristics in the India cohort. The top horizontal bar corresponds to cases (red) and controls (blue). The side bar plot shows the Spearman correlation value measuring the association between INLS3 and RAB20 expression and CD8+ and CD3+ cell count; green bars indicate a significant association. The lower horizontal bars correspond to female (orange) vs. male (green) followed presence (red) vs. absence (light gray) of participant characteristics; dark gray indicates no information available. The bottom bar plots show significant (green vertical bars) and non-significant (gray vertical bars) correlations with participant characteristics, including log_2_ HIV viral load; CD4+/CD8+/CD3+ cell counts, body mass index (BMI) and age.

Among the India cohort, CD8+ and CD3+ cell counts were significantly lower in cases than controls (Supplementary Figures 3B–D). Comparing Decision-tree gene expression to clinical variables, Spearman correlation values indicate a significant negative correlation between *INSL3* expression and both CD8+ and CD3+ cell counts. No cluster was associated with clinical variables (Smoke, Cough, Cavitation, Death, Viral load, CD4, Age or BMI) (Figure 3).

### Validation of the Decision-Tree Signature

**Figure 4:**
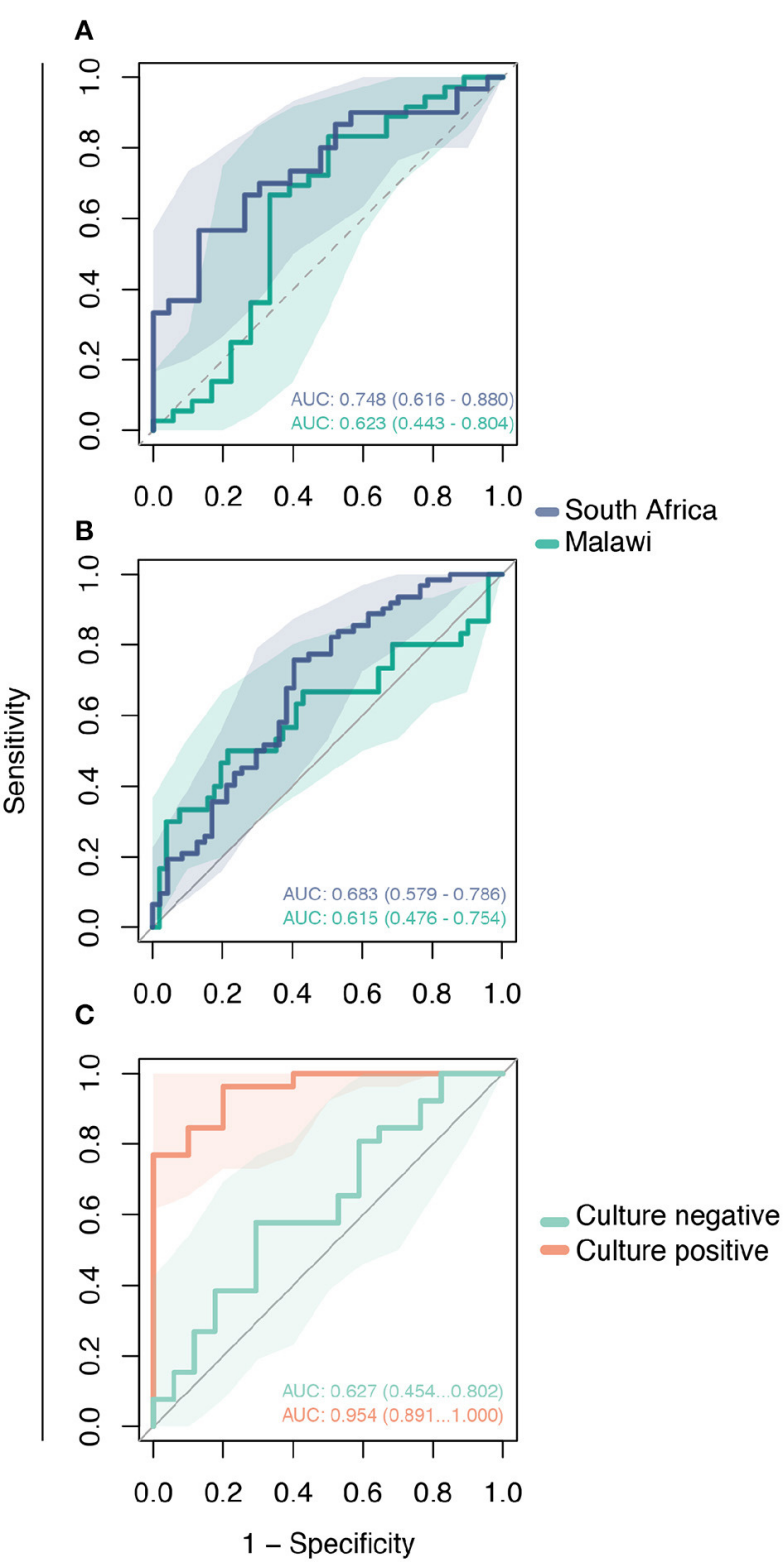
Validation of the Decision-tree gene signature using publicly available microarray datasets. Receiving operating characteristic curve analysis evaluating the performance of the 2-gene signature to distinguish comparison groups in the: **(A)** GSE39940 dataset—children living with HIV from South Africa and Malawi coinfected with TB or other respiratory diseases (ORD) (HIV-TB vs. HIV-ORD); **(B)** GSE37250 dataset—adults living with HIV from South Africa and Malawi coinfected with TB or ORD (HIV-TB vs. HIV-ORD); and **(C)** GSE39939 dataset from Kenya—patients with HIV-TB co-infection with and without culture-confirmed TB (culture-positive vs. culture-negative).

We performed ROC analysis to determine the sensitivity of the 2-gene signature to distinguish active TB among three validation cohorts. As shown in Figure 4, the Decision-tree signature performed best among South African cohorts with AUC ranging between 0.683 and 0.748; performance was lower among Malawi cohorts with AUC ranging between 0.615 and 0.623 (Figures 4A,B). The 2-gene signature demonstrated high accuracy to predict active TB with an AUC of 0.945 for distinguishing culture-confirmed TB from culture-negative TB (Figure 4C).

## Discussion

Transcriptomic signatures for TB diagnosis have been previously identified using various approaches, including differentially expressed genes, pathway analysis and subsetting genes associated to symptomatology (15, 16, 31). Although the blood transcriptomic profiling can improve diagnosis and understanding of TB infection, population-specific gene expression could interfere with performance across different regions (32). This study identified a 2-gene parsimonious signature that accurately classified active TB among people with advanced HIV infection in two geographically distinct cohorts. More importantly, the signature fared well to distinguish active TB from latent tuberculosis infection (LTBI) as well as other respiratory diseases when applied to other African datasets. Finally, the signature performed best among those with culture-confirmed TB and is likely an indicator of mycobacterial replication, suggesting the potential to extrapolate its use for TB treatment monitoring.

The prediction of TB diagnosis in PLWH improved when Indian and Ugandan datasets were combined. The two genes generated by the machine learning algorithm (*RAB20* and *INSL3*) were able to accurately distinguish active TB from non-TB. *RAB20*, a member of the RAS Oncogene Family, is involved in the maturation and acidification of phagosomes. More specifically, *RAB20* regulates the endosomal membrane, thus playing an important role in phagosome integrity and control of *Mycobacterium tuberculosis* (*Mtb*) replication in infected macrophages (33). This mechanism is also regulated by IFN-γ, assisting with *Mtb* infection control in macrophages (34). In contrast, *INSL3* is part of an insulin-like hormone superfamily and is associated with human testicular cell tumors (35), but has not been previously associated with TB infection or disease. Notably, the strong negative correlation observed between *INSL3* expression and CD8+/CD3+ cell count (rho−0.6) suggests a significant role in immune cell regulation among PLWH with active TB from India. The influence of *INSL3* on CD8+ and CD3+ cells could be associated with its regulation of *TIMP2* (36), a member of the NF-KappaB Family Pathway.

Although the 2-gene signature performed well in both discovery cohorts, we observed considerable geographic differences in gene expression between India and Uganda. Specifically, samples from Africa presented more DEGs (528 genes) than India (77 genes), and only 40 common DEGs were identified across the sites. A multitude of factors alter the immune response and may explain the observed differences, including ethnic population, dietary, environmental and seasonal differences (37, 38). Variable performance of TB signatures in Indian and African discovery cohorts provides additional evidence of population-specific gene expression. The performance of TB signatures varied with lower AUC observed among India samples compared to Uganda. Even signatures proposed among PLWH, such as Esmail_82, Esmail_203, Esmail_893 (31), Kaforou_27, Kaforou_OD_44, Kaforou_OD_53 (28), Sambarey_HIV_10 (39), and Rajan_HIV_5 (40), demonstrated differential performance among Indian and Ugandan cohorts. The total number of genes varies widely across signatures, ranging from 5 to 893 genes, and could explain the differential performance (AUC) in classifying TB status among PLWH, but also suggests a possible population bias in each signature that could interfere with its use in other geographic locations.

The differential gene expressions observed between Indian and Ugandan cohorts was not unexpected. Despite the differences, however, the discovery cohorts shared 40 differentially expressed genes for TB, and two important pathways were found to be upregulated in both cohorts (18). The Toll-like receptor cascade pathway has been previously associated with TB and HIV, indicating the role of *Mtb* in the regulation of HIV replication (41). The neutrophil degranulation pathway has also been associated with TB, but the exact role of neutrophils remains ambiguous with potential to be associated with *Mtb* clearance as well as increased disease severity and mortality (42). Overall, these pathways suggest that TB disease may influence peripheral blood mononuclear cell expression in PLWH.

The performance of the novel 2-gene signature is heterogeneous in the external validation data sets, but the 2-gene signature has fair overall accuracy to distinguish TB. Accuracy ranged from 0.683 to 0.748 in the African cohort comprising children and adults, and inferior performance was observed in the Malawi cohort with AUC values ranging from 0.615 to 0.623. The difference in performance suggests that population-associated gene expression interferes with TB classification in PLWH. Despite the unsatisfactory performance of the 2-gene signature in these data, some aspects should be accounted. In this dataset, the control group was composed of PLWH and other respiratory diseases. The control group composition and population bias may have contributed to reduced AUC values. Interestingly, TB classification accuracy was high for patients with culture-confirmed TB in the Kenya cohort (AUC 0.954) while reduced performance was observed among patients without culture-confirmed TB (AUC 0.627). This finding suggests an association of the two-gene signature with bacterial load and that longitudinal change in expression of this gene signature could also be used to monitor bacillary load in response to treatment.

Gene signatures derived from multiple cohorts were validated using a targeted approach, reverse transcriptase multiplex ligation-dependent probe amplification (RT-MLPA) in a multisite study that comprised cohorts with and without HIV. The analysis revealed FCGR1A [high-affinity IgG Fc receptor 1 (CD64)] as a consistent single-gene classifier of active TB disease, in the presence and absence of HIV (43). FcGR1A was also reported to function as a consistent single gene classifier of active TB even in advanced HIV in the Uganda cohort included in this study (18). In an Ethiopian cohort, five genes (CD8A, TIMP2, CCL22, FCGR1A, and TNFRSF1A), were shown to segregate active TB from non-active TB in HIV patients (44). In another study, also in an Ethiopian cohort of HIV co-infected TB patients, 7 genes (FCGR1A, RAB24, TLR1, TLR4, MMP9, NLRC4, and IL1B) accurately discriminated between active tuberculosis disease and latent infection (45). RISK6 is a prognostic signature derived from baseline blood samples in a SA adolescent cohort of progressors and non-progressors (27). The signature is an aggregate of nine transcript pairs that was derived by separately linking each of three transcripts upregulated in progressors (GBP2, FCGR1B, and SERPING1), to three transcripts downregulated in progressors (TUBGCP6, TRMT2A, and SDR39U1), relative to non-progressors. RISK6 also performed well in diagnosing active TB in HIV-uninfected and HIV-infected persons (27). Of note, none of the studies included cohorts from India. Additional head-to-head comparative studies in larger cohorts are needed to determine whether the 2-gene signature reported here works across ethnicities and comorbidities, including HIV. Furthermore, whether the same gene signatures will perform well in segregating TB from HIV with differing CD4 counts and differing peripheral inflammation also needs to be determined.

Despite yielding interesting results, our study has some limitations. First, the sampling size is not ideal, with 25 samples from India and 33 from Uganda, and has resulted in more variability observed in the study. Second, the metadata from all validation datasets do not have the CD4 count value for each patient, but the overall cohort data report much higher CD4 value than our cohort. This may have contributed to reduction in performance of our signature. For clinical application, more studies are required to standardize a gene expression-based protocol. Furthermore, RNA seq-based signatures need to be further developed for use in clinical practice to distinguish PLWH with TB from those with LTBI or other respiratory diseases.

In conclusion, despite populational-specific differential gene expression, the *RAB20* and *INSL3* genes outperformed all previously proposed TB signatures to accurately distinguish TB from non-TB among multiple cohorts from different geographical regions. This parsimonious 2-gene signature also performed well among those with culture-positive TB, indicating its potential use for TB treatment monitoring. Our study provides evidence supporting a promising, novel and non-sputum-based biomarker for TB diagnosis, especially for those with advanced HIV infection in whom TB diagnosis is often difficult with sputum-based diagnostics. Future studies are needed to confirm our findings.

## Data Availability Statement

The datasets presented in this study can be found in online repositories. The names of the repository and accession number can be found here: GEO database (Accession number GSE162164, https://www.ncbi.nlm.nih.gov/geo/query/acc.cgi?acc=GSE162164).

## Ethics Statement

The studies involving human participants were reviewed and approved by (1) Byramjee Jeejeebhoy Government Medical College Clinical Trials Unit. (2) INI-FIOCRUZ, Brazil; FMT, Brazil. (3) Johns Hopkins University School of Medicine, USA. (4) Boston Medical Campus (BUMC), USA. (5) Rutgers New Jersey Medical School IRB, USA. The patients/participants provided their written informed consent to participate in this study.

## Author Contributions

VM, DK, VR, PS, and JE contributed to study design. VK, AQ, and BA contributed toward data acquisition. PS, AQ, and BA contributed equally toward data interpretation. VK, SSan, AK, SSal, DK, and VM were responsible for patient recruitment, sample collection, storage, and analysis of clinical data. VK, AQ, VM, PS, and BA contributed equally to writing the manuscript. All authors read and approved the final manuscript.

## Conflict of Interest

The authors declare that the research was conducted in the absence of any commercial or financial relationships that could be construed as a potential conflict of interest.

## Supplementary Material

Supplementary Figure S1 **(A,B)** Volcano plots showing differentially expressed genes (DEG) for TB using whole-blood samples from India **(A)** and Uganda **(B)** cohorts. Red indicates DEGs, defined as change in gene expression with log_2_ Fold Change ±1.4 and FDR <0.05; green indicates change in gene expression with log_2_ Fold Change ±1.4; blue indicates change in gene expression with FDR <0.05; and gray indicates no significant change in gene expression. **(C)** The Venn diagram shows the number of site-specific and shared DEGS. **(D)** The Bar plot shows the log_2_ Fold Change of the 40 DEGs shared across study sites.

Supplementary Figure S2 Heatmap of the differentially expressed genes (DEG) for TB identified in the India **(A)** and Uganda **(B)** cohorts.

Supplementary Figure S3 The dot plot demonstrates the presence of Decision-tree genes (INSL3 and RAB20) in previously proposed TB gene expression signatures **(A)**. Boxplots show the associations of CD3 **(B)**, CD8 **(B)**, and CD4 **(C)** cell counts and HIV viral load **(D)** with TB-HIV co-infection status in the India cohort. Clinical variables were compared among cases (TB-HIV) and controls (HIV-only) using the Wilcoxon test. Only CD3 and CD8 cell counts were significantly associated with TB status.

Supplementary Table 1 Systematic literature review but restricted to the signatures present in TBSiginatureProfiler package, plus the RISK6 used as reference. Signature names represent the first author's name of the corresponding publication, suffixed with number of constituent genes that are present in the RNAseq dataset. Table includes number of genes, method, population, HIV status and treatment, geographical region, and participant condition. TB, tuberculosis; LTBI, latent tuberculosis infection; HHC, household contacts; SARC, sarcoidosis.

Supplementary Table 2 File with the differentially expressed genes (DEG) list from India, Uganda cohort, and the common genes. The log2 Fold Change, log2 Fold Change SE, p-value, and FDR are also provided for each gene.

## Abbreviations

ART: antiretroviral therapy
ATT: anti-tuberculosis treatment
AUC: area under the curve
DEG: differentially expressed genes
HIV: human immunodeficiency virus
TB: tuberculosis
PLWH: people living with HIV
PCA: principal component analysis.
