# Supplementary figures and images for: A Two-Gene Signature for Tuberculosis Diagnosis in Persons With Advanced HIV
Source: Front Immunol. 2021 Feb 22;12:631165. doi: 10.3389/fimmu.2021.631165 (PMC7937880; doi:10.3389/fimmu.2021.631165)

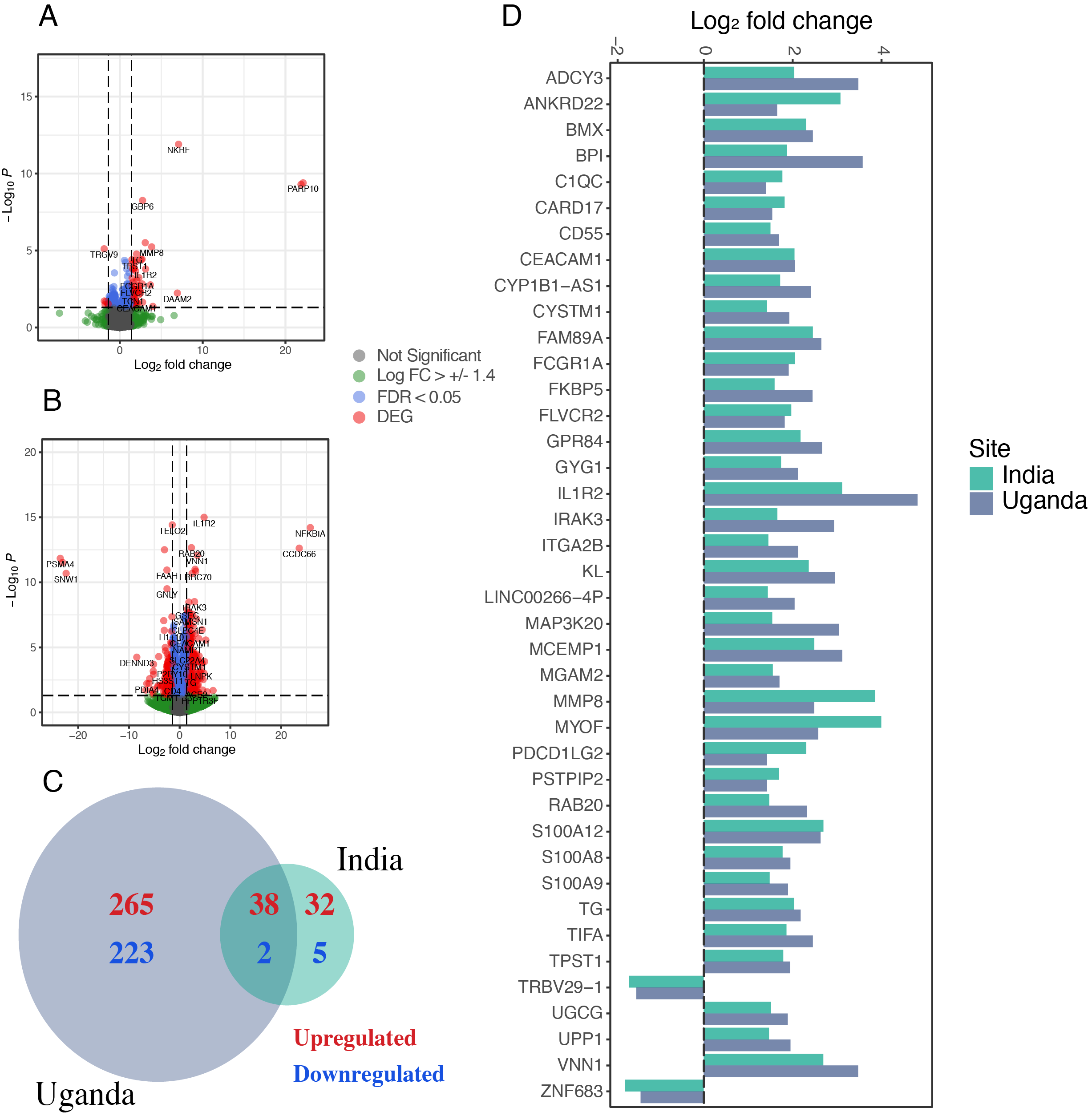

Supplement: Supplementary Figure S1 — (A,B) Volcano plots showing differentially expressed genes (DEG) for TB using whole-blood samples from India (A) and Uganda (B) cohorts. Red indicates DEGs, defined as change in gene expression with log2 Fold Change ±1.4 and FDR <0.05; green indicates change in gene expression with log2 Fold Change ±1.4; blue indicates change in gene expression with FDR <0.05; and gray indicates no significant change in gene expression. (C) The Venn diagram shows the number of site-specific and shared DEGS. (D) The Bar plot shows the log2 Fold Change of the 40 DEGs shared across study sites. [file Image_1.tif]

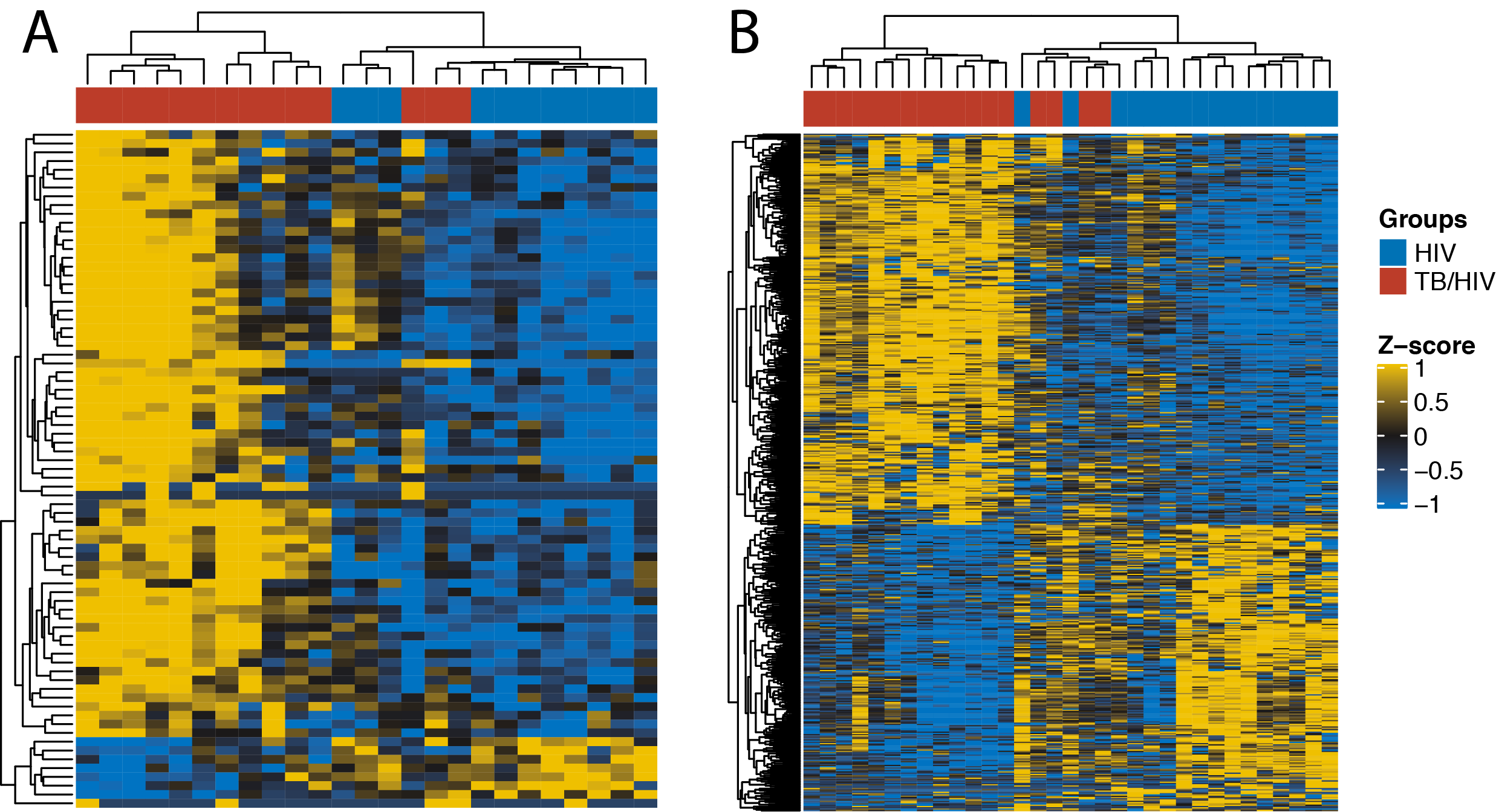

Supplement: Supplementary Figure S2 — Heatmap of the differentially expressed genes (DEG) for TB identified in the India (A) and Uganda (B) cohorts. [file Image_2.tif]

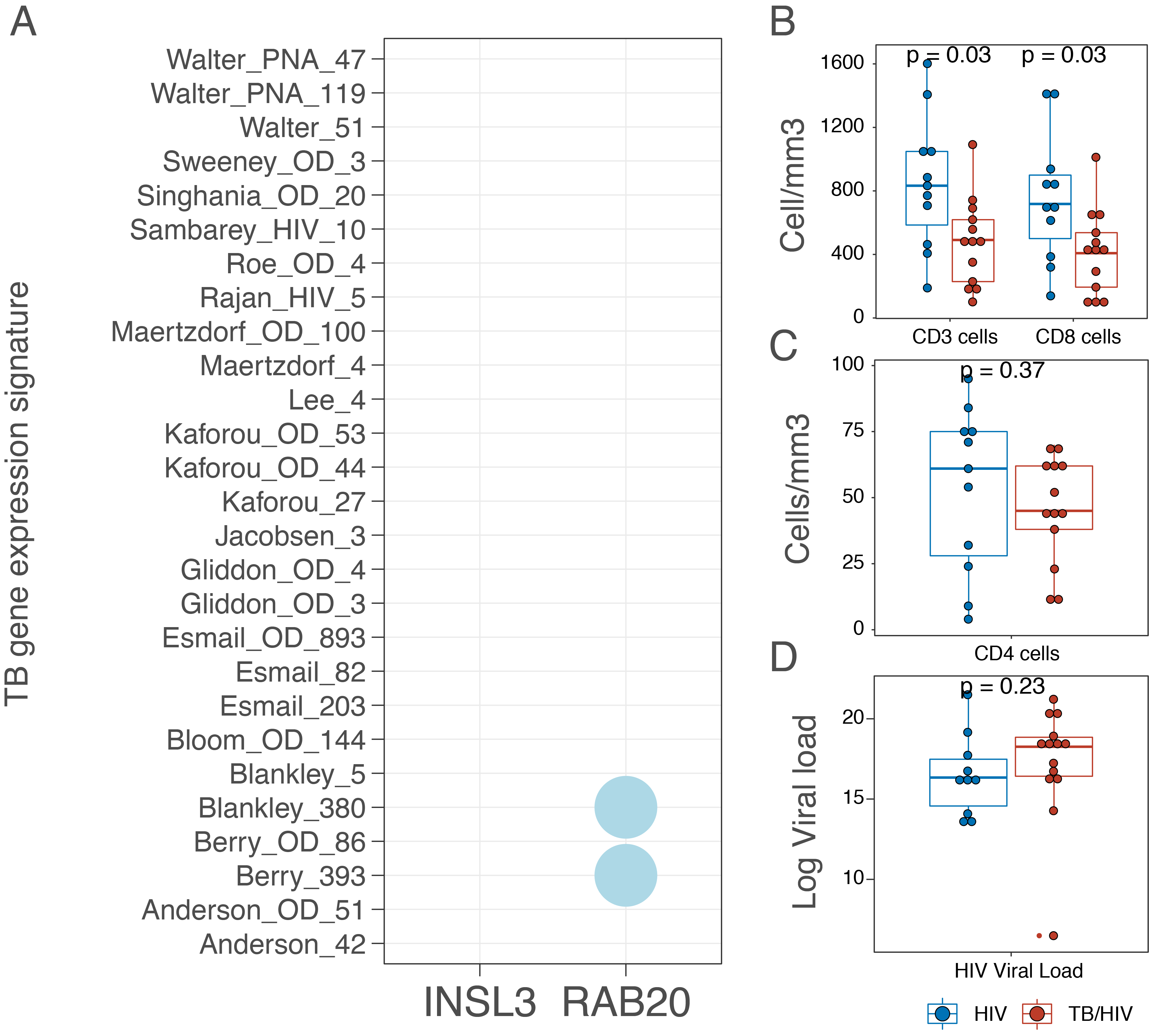

Supplement: Supplementary Figure S3 — The dot plot demonstrates the presence of Decision-tree genes (INSL3 and RAB20) in previously proposed TB gene expression signatures (A). Boxplots show the associations of CD3 (B), CD8 (B), and CD4 (C) cell counts and HIV viral load (D) with TB-HIV co-infection status in the India cohort. Clinical variables were compared among cases (TB-HIV) and controls (HIV-only) using the Wilcoxon test. Only CD3 and CD8 cell counts were significantly associated with TB status. [file Image_3.tif]
